# The EGF Repeat-Specific O-GlcNAc-Transferase Eogt Interacts with Notch Signaling and Pyrimidine Metabolism Pathways in *Drosophila*

**DOI:** 10.1371/journal.pone.0062835

**Published:** 2013-05-09

**Authors:** Reto Müller, Andreas Jenny, Pamela Stanley

**Affiliations:** 1 Department of Cell Biology, Albert Einstein College of Medicine, New York, New York, United States of America; 2 Department of Developmental and Molecular Biology, Albert Einstein College of Medicine, New York, New York, United States of America; 3 Department of Genetics, Albert Einstein College of Medicine, New York, New York, United States of America; University of Dayton, United States of America

## Abstract

The O-GlcNAc transferase Eogt modifies EGF repeats in proteins that transit the secretory pathway, including Dumpy and Notch. In this paper, we show that the Notch ligands Delta and Serrate are also substrates of Eogt, that mutation of a putative UDP-GlcNAc binding DXD motif greatly reduces enzyme activity, and that Eogt and the cytoplasmic O-GlcNAc transferase Ogt have distinct substrates in *Drosophila* larvae. Loss of Eogt is larval lethal and disrupts Dumpy functions, but does not obviously perturb Notch signaling. To identify novel genetic interactions with *eogt*, we investigated dominant modification of wing blister formation caused by knock-down of *eogt.* Unexpectedly, heterozygosity for several members of the canonical Notch signaling pathway suppressed wing blister formation. And importantly, extensive genetic interactions with mutants in pyrimidine metabolism were identified. Removal of pyrimidine synthesis alleles suppressed wing blister formation, while removal of uracil catabolism alleles was synthetic lethal with *eogt* knock-down. Therefore, Eogt may regulate protein functions by O-GlcNAc modification of their EGF repeats, and cellular metabolism by affecting pyrimidine synthesis and catabolism. We propose that *eogt* knock-down in the wing leads to metabolic and signaling perturbations that increase cytosolic uracil levels, thereby causing wing blister formation.

## Introduction

Glycosylation is the most common post-translational modification of proteins. Different classes of glycans or individual sugars within a glycan have been shown to regulate cell-cell recognition, cell migration, cell proliferation, the binding of pathogenic viruses and bacteria, growth factor and cytokine signaling, and Notch signaling [1]. Most sugars are transferred to proteins within the secretory pathway. For example, Fuc, Man, Glc, Xyl, GalNAc and GlcNAc may be transferred to Ser or Thr, and subsequently substituted with additional sugars to generate O-glycans. O-glycans, as well as single sugar residues, may confer a variety of functions on glycoproteins. For example, in *Drosophila*, Fringe adds a GlcNAc to O-Fuc on Notch (N) epidermal growth factor-like (EGF) repeats, thereby altering the binding of Notch ligands Delta (Dl) and Serrate (Ser), and up- or down-regulating Notch signaling, respectively [2], [3]. This is critical for controling Notch signaling during boundary formation in the wing imaginal disc, in leg development, and in the eye [4], [5]. Another important protein modification is the addition of O-GlcNAc to cytoplasmic and nuclear proteins, the only glycosylation reaction known to occur outside the secretory pathway in vertebrates [6]. In *Drosophila,* cytosolic O-GlcNAc regulates the activities of cytoskeletal proteins, transcription factors and enzymes. It also links metabolism to epigenetics through histone modification [7], and is an efficient UDP-GlcNAc/nutrient sensor [8]. *Drosophila* Ogt is encoded by the *super sexcomb (sxc)* gene, and *sxc* null mutants are late pupal lethal [9].

The addition of O-GlcNAc to proteins sequestered within the secretory pathway was first reported as a modification of *Drosophila* Notch EGF repeat 20 (EGF20) [10]. The enzyme that catalyzes this transfer was subsequently identified as an EGF repeat-specific O-GlcNAc transferase and termed Eogt [11]. Eogt is resident in the endoplasmic reticulum (ER) [11] and generates β-linked GlcNAc on Ser or Thr of EGF repeats in the consensus sequence C_5_XXGXT/SGXXC_6_ [12]. *Drosophila* Notch has 17 EGF repeats with this consensus site. Dumpy (Dp), a transmembrane protein largely in the extracellular matrix without a clear mammalian homologue [13], has 86 of 300 EGF repeats with this consensus sequence, and is a substrate of Eogt [11]. In this paper, we report Dl and Ser as additional substrates of Eogt in *Drosophila.*

Loss of *eogt* and thus O-GlcNAc on Dumpy, affects apical extracellular matrix (aECM)/cuticle functions mediated by Dumpy in larvae [11]. Homozygous *eogt* mutants die at around larval stage 2, and phenocopy lethal *dp* mutants [11]. Loss of function mutations of *dp* or *eogt* in the adult wing result in blistering [11], [14]. Several other loci are known to genetically interact with *dp* [15]. For example, *dp* cooperates with genes encoding the Zona Pellucida domain proteins *papillote* (*pot*) and *piopio* (*pio*) in maintaining the structural integrity of the aECM, by mediating its attachment to the epidermis in the pupal wing, and in anchoring the aECM to the transalar array, the cytoskeletal and junctional support important in integrin-mediated basal adhesion [16]. Furthermore, *dp* mutants genetically interact with mutants of pyrimidine metabolism [17]–[19].

To identify novel genes that interact with *eogt*, we performed dominant genetic interaction assays of an *eogt* RNAi-induced wing phenotype. Unexpectedly, even though *eogt* mutants do not show an *N* phenotype [11], reduction of *N* activity by removing one allele of various pathway members, including the transcription regulators *Suppressor of Hairless* (*Su(H)*) and *mastermind* (*mam*), strongly suppressed the wing blister phenotype caused by loss of *eogt*. In addition, loss of one allele of genes encoding enzymes of the pyrimidine pathway that produce uridine nucleosides and UDP-GlcNAc, suppressed the *eogt* RNAi phenotype, while loss of alleles of uracil catabolic enzymes enhanced it. We propose that uracil toxicity, previously implicated in causing wing blisters in *dp;su(r)* double mutants [19], is a likely basis of the blistering phenotype observed in the absence of *eogt*.

## Results

### Human EOGT has a DXD Motif Important for Catalysis

**Figure 1:**
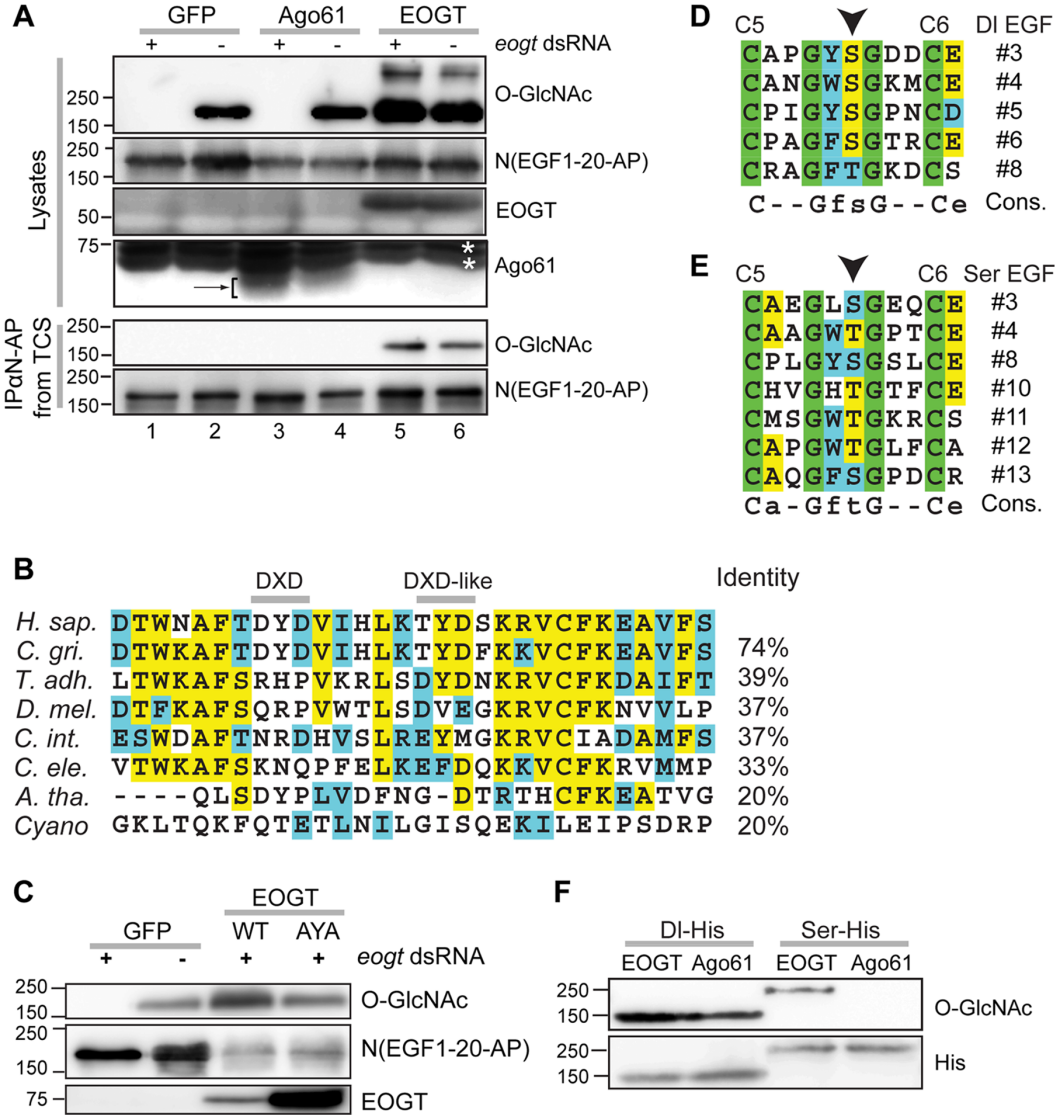
Human EOGT requires a DXD motif for optimal activity. (A) Human EOGT is active in S2 cells. Western blots of lysates (top) and immunoprecipitates from conditioned medium (bottom) from S2 cells treated with (+) or without (−) dsRNA against *eogt,* and transfected with *N(EGF1-20)-AP* and *GFP*, *Ago61* or *EOGT*, as noted. The target of each antibody is shown on the right. Arrow identifies Ago61 band; *identifies non-specific bands. (B) Alignment of putative catalytic regions of Eogt homologs. % identity with the full-length human protein is shown on the right. *C. gri*: *Cricetulus griseus*, *T. adh*.: *Trichoplax adhaerens*, *C. int*.: *Ciona intestinalis.* (C) Western analysis of lysates from S2 cells treated with *eogt* dsRNA as noted, and transfected with *GFP*, *EOGT* wild-type (DYD), or *EOGT* mutant (AYA) cDNA. Mutant *EOGT*(AYA) was less active even though consistently expressed at much higher levels. (D, E) The proposed Eogt consensus site is present in several EGF repeats of *Drosophila* Dl (D) and Ser (E). (F) Western analysis of lysates from S2 cells transfected with soluble extracellular domain of His-tagged Dl (Dl-His) or Ser (Ser-His) and either *EOGT* or *Ago61* cDNA as indicated.

We previously identified *Drosophila CG9867* as a putative glycosyltransferase gene required for viability of *Drosophila melanogaster* [20]. This gene was recently shown to encode Eogt [11], and to be conserved in mouse [21]. We now show that one of the splice forms of the human gene *C3orf64* cloned from HEK 293T cells (GenBank accesssion number KC347596.1), is an active human EOGT homologue. To establish that it has EOGT activity, *Drosophila* S2 cells or S2 cells incubated with a dsRNA designed to target endogenous *eogt,* were co-transfected with a soluble *Drosophila* Notch fragment tagged at the C-terminus with alkaline phosphatase (N(EGF1-20)-AP). There are 17 perfect matches to the consensus site for Eogt modification on *Drosophila* Notch (EGF repeats 3, 5, 9, 11–17, 19, 20, 22, 25–28). N(EGF1-20)-AP was transfected together with either a plasmid containing GFP, or the human *EOGT* cDNA, or a cDNA of mouse *Ago61* as negative control. Ago61 is a putative glycosyltransferase from the same family as *eogt* in the Carbohydrate Active enZymes (CAZy) database [22]. N(EGF1-20)-AP was immunoprecipitated from conditioned medium using anti-AP beads and cell lysates were prepared. Each lysate contained a comparable amount of N(EGF1-20)-AP based on detection with anti-AP antibody (Ab), and EOGT and Ago61 were present in the appropriate samples (Fig. 1A). Detection of O-GlcNAc on N(EGF1-20)-AP by anti-β-O-GlcNAc antibody CTD110.6 [23] showed that *eogt* dsRNA treatment of S2 cells abolished O-GlcNAcylation of N(EGF1-20)-AP by endogenous Eogt compared to untreated cells (Fig. 1A, lanes 1, 2). Co-transfection of mouse *Ago61*, did not result in an O-GlcNAc signal in the presence of *eogt* dsRNA, nor did it increase the signal in untreated cells (Fig. 1A, lanes 3, 4). In contrast, exogenous human *EOGT* cDNA, resistant to the *Drosophila eogt* dsRNA, strongly O-GlcNAcylated N(EGF1-20)-AP (Fig. 1A, lanes 5, 6). O-GlcNAc-positive bands of molecular weight higher than N(EGF1-20)-AP and not detected by anti-AP Ab, were observed in cells co-transfected with *EOGT* (Fig. 1A and not shown). O-GlcNAc was detected on N(EGF1-20)-AP immunoprecipitated from conditioned medium only when *EOGT* was overexpressed (Fig. 1A).

*Drosophila* and mouse Eogt are metal-dependent glycosyltransferases [11], [21], that often contain a DXD motif implicated in metal coordination of the nucleotide sugar donor [24]. Mutation of DXD motifs diminishes or abolishes transferase activity [25]. Vertebrate EOGT proteins have a potential DXD motif (DYD), which is highly conserved among vertebrates, while invertebrates have a related motif at 6 aa towards the C-terminus (Fig. 1B). We mutated DYD in human EOGT to AYA, thereby eliminating the side chains predicted to coordinate bivalent cations. Wild-type *EOGT*(DYD) and mutant *EOGT*(AYA) were transfected into S2 cells treated with *eogt* dsRNA. Compared to wild-type, mutant *EOGT*(AYA) was considerably less efficient at generating an O-GlcNAc signal on co-transfected N(EGF1-20)-AP, even though expression of the mutated protein was reproducibly much higher (Fig. 1C). The low residual activity of the mutant indicates that the DXD motif is not absolutely required for EOGT function, consistent with the observation that low levels of *Drosophila* Eogt activity were detected *in vitro* in the absence of divalent cations [11].

Several protein sequences in *Drosophila* other than Notch contain the EOGT recognition consensus sequence C_5_XXGXT/SGXXC_6_ in EGF repeats [11], [12]. For example, Notch ligands Dl and Ser contain five and seven perfect matches, respectively (Fig. 1D and 1E), and their ability to be modified with O-GlcNAc by Eogt in S2 cells was tested. Dl was readily modified with O-GlcNAc by endogenous Eogt in S2 cells, and the modification was increased by co-transfection with human *EOGT*. While O-GlcNAc was not detected on Ser exposed to endogenous Eogt in S2 cells, co-expression of human *EOGT* resulted in O-GlcNAcylation of Ser (Fig. 1F).

### *Drosophila* Eogt has Predominantly High Molecular Weight Targets in Larvae

**Figure 2:**
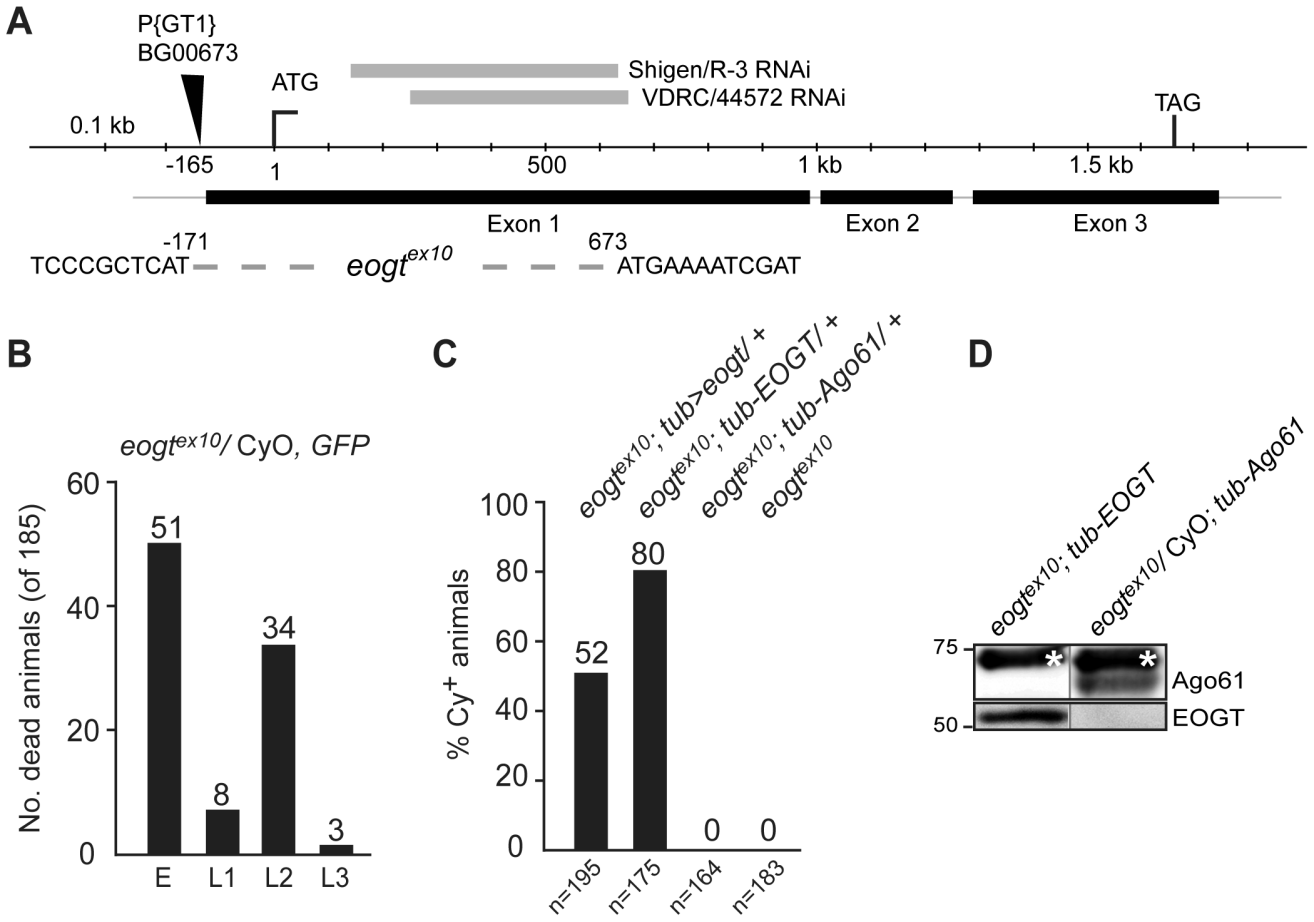
Human *EOGT* can substitute for *Drosophila eogt in vivo.* (A) Schematic of the *eogt* locus. The region deleted in *eogt* ^ex10^ by imprecise excision of P-element *BG00673* is indicated by a dotted line and flanking sequences. Coordinates of the deletion are given relative to the start codon. The deletion removed the start codon and 224 aa of the coding region. Locations of the dsRNA Shigen/R-3 and VDRC/44572 are shown as grey bars on top. (B) Homozygous *eogt^ex10^* mutants die in L2. Number of dead offspring at indicated stages of an *eogt* ^ex10^/CyO, *twi-GFP* strain. Dead embryos expressed GFP and were probably homozygous for the balancer. Animals dying in L1-L3 did not express GFP and were thus homozygous for *eogt* ^ex10^. All animals that survived to adulthood were heterozygotes carrying CyO (n = 89). (C) *Drosophila eogt* (*tub*>*eogt*) and human *EOGT* (*tub-EOGT*), but not mouse *Ago61* (*tub*-*Ago61*), rescued *eogt* ^ex10^ animals that obtained a transgene. (D) Western blots of adult fly lysates confirm that human EOGT and Ago61 were expressed in the stocks assessed for rescue in (B). *non-specific band.

To assess substrates of Eogt *in vivo*, an *eogt* mutant was made using imprecise excision of a nearby P element and mapped by PCR and sequencing. Excision *eogt^ex10^* lacks the first 0.8 kb of exon 1, which includes the start codon (Fig. 2A). Using a GFP-marked second chromosome balancer, the *eogt^ex10^* allele was shown to be lethal at larval instar L2 (Fig. 2B), a stage at which the larvae can remain for up to 48 hours before dying, with a few escapers reaching L3. All surviving adults were CyO (n = 89), showing that no *eogt^ex10^* homozygotes survived. Homozygous *eogt^ex10^* mutants were rescued to adulthood upon ubiquitous expression of *Drosophila UAS*-*eogt* under the control of *tubulin-Gal4* (*tub-Gal4>UAS*-*eogt*, from here on termed *tub>eogt*; Fig. 2C). Rescue attempts with *heat-shock*-*Gal4*- or *actin-Gal4*-driven *UAS*-*eogt* were unsuccessful (not shown). Furthermore, the human ortholog *EOGT* driven by the *tubulin* promoter (*tub*-*EOGT*) also rescued *Drosophila eogt^ex10^* lethality (Fig. 2C and 2D). Rescued animals showed no visible phenotypes. In contrast, mouse *Ago61* driven by the tubulin promoter (*tub*-*Ago61*) did not rescue *eogt^ex10^* (Fig. 2C), even though it was robustly expressed (Fig. 2D).

**Figure 3:**
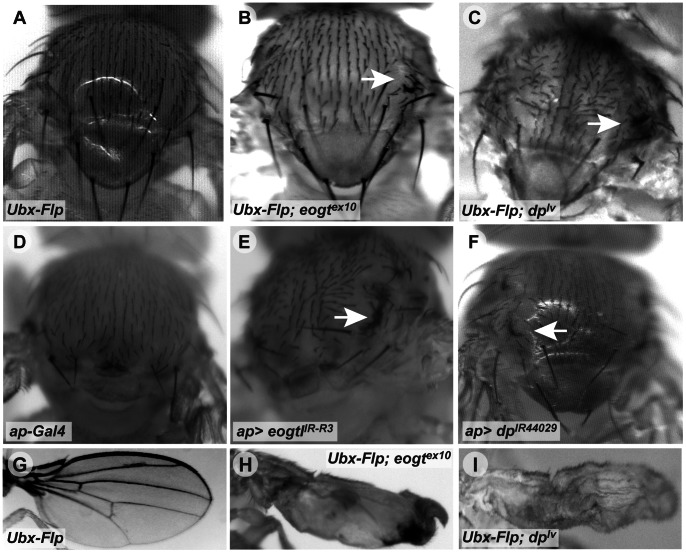
Loss of *eogt* phenocopies loss of *dp in vivo*. (A–C) Compared to control clones (A), *Ubx-Flp*-induced *eogt^ex10^* clones (B) caused vortex phenotypes on the thorax (arrow), similar to *dp^lv^* clones (arrow in C). (D–F) *ap-Gal4*-mediated RNAi knock-down of *eogt* phenocopied the vortex phenotype of *eogt* mutant clones (arrow in E), similar to *dp* knock-down (arrow in F). Note that *ap-Gal4* alone had a different bristle phenotype (D). (G–I) Compared to control clones (G), *eogt^ex10^* (H) and *dp^lv^* clones (I) caused deformed wings with blisters.

In order to analyze *eogt^ex10^* mutant phenotypes and to compare them with *dp* mutants [26], *eogt^ex10^* and a lethal allele of *dp* (*dp^lv^*) were recombined onto Frt40A chromosomes, and the consequences of loss of *eogt* or *dp* in clones of homozygous mutant cells induced by *Ubx-Flp* [27] were analyzed. Mutant clones of *eogt* ^ex10^ or *dp^lv^* in the thorax caused the formation of vortices in both cases, whereas none were observed in control clones (Fig. 3A–3C). Consistently, knock-down of *eogt* using RNAi under control of the *apterous* promoter (*ap-Gal4*) led to disorganization of thoracic bristles and a vortex phenotype (not shown), as well as a comma phenotype in *ap-Gal4*>*eogt^IR-R3^* (Fig. 3E). Targeted knock-down of *dp* in the thorax by RNAi (*ap-Gal4>dp^IR44029^*) also caused vortices to develop (Fig. 3F). However, the penetrance of this *dp* vortex phenotype was weak at 31°C, even when a *dicer2* transgene (*UAS*-*dcr2*) was included to increase knock-down efficiency. Importantly, unmarked mutant *eogt* ^ex10^ clones in the wing resulted in a severely deformed wing with blisters, similar to the phenotype observed in *dp^lv^* clones (Fig. 3G–3I).

**Figure 4:**
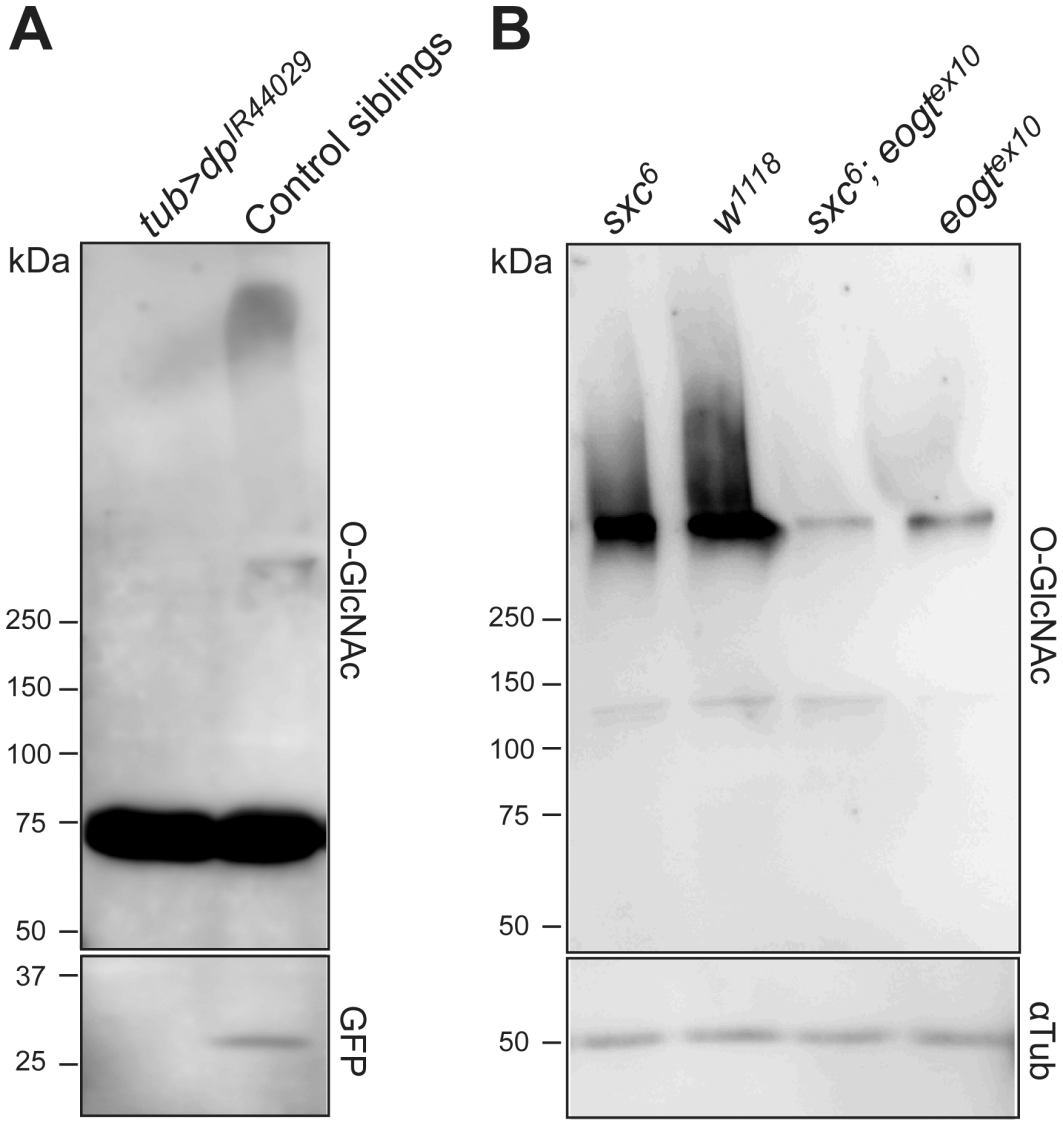
Molecular targets of Eogt. (A) Western analysis using mAb CTD110.6 of early pupal lysates of *tub*>*dp* ^IR^ and control siblings. An ∼75 kDa band served as loading control. The GFP signal in the lower panel confirmed the respective genotypes (i.e. all *dp* knock-down pupae contained *tub-Gal4* and not the TM3, *Ser*, *act*-*GFP* balancer over which it was kept). (B) Western analysis of control *w^1118^* and *ogt* mutant *sxc^6^*, *eogt^ex10^* and *sxc^6^, eogt^ex10^* double mutant L2 larval extracts to detect O-GlcNAcylated proteins using mAb CTD110.6 (upper panel). α−Tubulin was used as loading control (lower panel).

To assess Eogt modification of Dp, we used a *dp*-targeted RNAi construct that caused the expected *dp^oblique^* (*dp^o^*) phenotype when expressed at 18°C in the wing under *en-Gal4* (not shown). RNAi knock-down of *dp* under the strong and ubiquitously expressed *tub-Gal4* promoter at 31°C was late pupal lethal, precluding analysis at this stage. At 2^nd^ instar, larval lysates showed no difference in O-GlcNAc signal between *dp* knock-down and control (not shown), presumably reflecting maternal contribution of *dp*. However, in early pupal control lysates (GFP-positive), a high molecular weight O-GlcNAc signal was observed that was absent from *dp* knock-down pupal lysates, suggesting that Dp is a major target of Eogt (Fig. 4A). An additional O-GlcNAc signal of ∼75 kDa that was detected at stages beyond late L3, served as a loading control (Fig. 4A).

In order to identify general targets of Eogt in larval extracts, it was important to differentiate between targets of Ogt, which transfers O-GlcNAc to proteins of the cytoplasm and nucleus, and Eogt, which acts only on proteins that traverse the secretory pathway. We therefore recombined the Ogt mutant allele *sxc^6^* [9] and the *eogt^ex10^* mutant allele to obtain double mutants, for comparisons with *sxc^6^* or *eogt^ex10^* mutants and wild-type. Few O-GlcNAc-positive bands were detected in lysates of control or mutant L2 larvae under conditions optimized for detection of O-GlcNAc on EGF repeats (Fig. 4B). However, at molecular weights >250 kDa, control extracts gave a broad smear that aggregated at the interface of the stacking and running gels. There was a small reduction of O-GlcNAc signal in zygotic *sxc^6^* larvae compared to wild-type, which probably reflects loss of O-GlcNAc from intracellular substrates of Ogt including the cytosolic domains of membrane glycoproteins. By contrast, there was a very strong reduction of the O-GlcNAc signal in zygotic *eogt^ex10^* mutants, and a slightly lower signal in *sxc^6^/eogt^ex10^* double mutant lysates. The signal remaining in double mutant lysates is probably due to O-GlcNAc added by maternally provided Eogt or Ogt. Therefore, most O-GlcNAcylated proteins of >250 kDa, including *dp*, are substrates of Eogt.

### *Eogt* Knock-down is Sensitive to Dominant Modification

To identify additional pathways affected by Eogt, we investigated RNAi-mediated *eogt* knock-down phenotypes for use in genetic interaction analyses. Ubiquitous expression of two overlapping RNAi lines VDRC/44572 and Shigen/R-3 (Fig. 2A) under the control of the *tubulin* promoter at 30°C, was late pupal lethal. When RNAi expression was driven by *act-Gal4* at 30°C, 94% of the predicted VDRC/44572 animals hatched (n = 248), 37% of VDRC/44572 animals with a *UAS*-*dcr2* transgene hatched (n = 136), and no Shigen/R-3 animals (n = 162) were found. Knock-down of *eogt* using *en-Gal4* expressed in the posterior compartment, or *ap-Gal4* expressed in the dorsal compartment, induced blistering of the wing.

**Figure 5:**
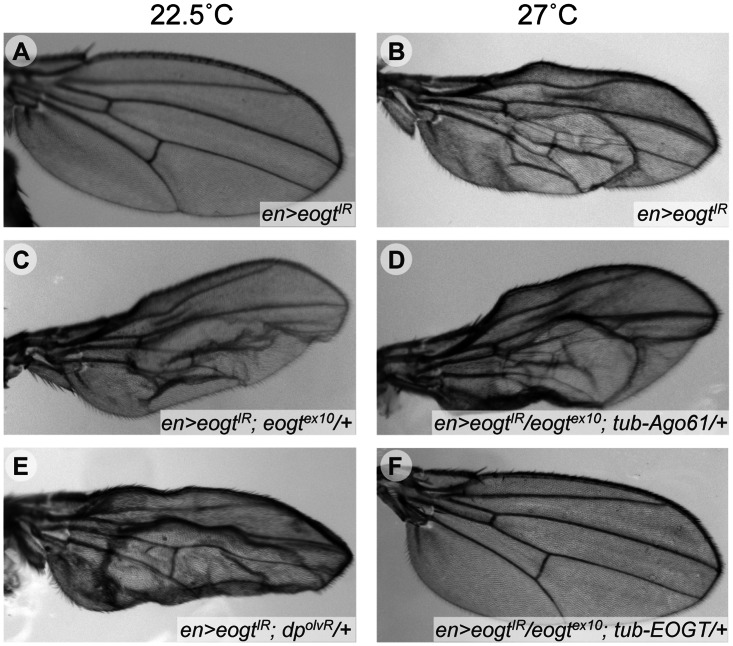
Temperature-sensitive wing blister assay for *eogt* interactors. Wings of flies raised at 22.5°C (A, C, E) or 27°C (B, D, F). *en*>*eogt^IR^* wings were normal at 22.5°C (A) but blistered in the posterior compartment at 27°C (B). At the low temperature, blistering was induced when one gene dose of *eogt* (*eogt^ex10^*/+) or *dp* (*dp^olvR^*) was removed (C and E, respectively). While blistering at the higher temperature was not affected by co-expression of *Ago61* (D), it was suppressed by co-expression of human *EOGT* (F), even when one gene dose of *eogt* had been removed.

To establish genetic interaction assays, we tested whether the wing blister phenotype of *eogt* provided a sensitive baseline to identify dominant modifiers. An *en-Gal4*-driven RNAi knock-down with *Dicer* (*en-Gal4*, *UAS*-*VDRC^44572^*, *UAS*-*dcr2*; from here on designated *en*>*eogt^IR^*), caused the formation of wing blisters specifically within the posterior compartment. No blisters were seen in the anterior compartment. The phenotype was temperature sensitive with virtually complete penetrance at 27.0°C, while hardly any blisters were found at 22.5°C (Fig. 5A and 5B and Table 1). Importantly, an *eogt^ex10^* heterozygous background enhanced the frequency of flies with wing blisters up to 30% at 22.5°C (Fig. 5C and Table 1), while co-expression of human *EOGT* completely reverted the blister phenotype at 27°C, even in the absence of one gene dose of *eogt* (Fig. 5F and Table 1). Co-expression of *Ago61* did not suppress blisters (Fig. 5D), consistent with biochemical data (Fig. 1).

**Table 1:** Specificity of *en*>*eogt^IR^* Interactions.

Allele	% Flies with Blisters (22.5°C)	% Flies with Blisters (27°C)
*w^1118#^*	**0** (0/161)	**100** (212/212)
*w^1118#^*	**0** (0/125)	**95** (300/316)
*P-BG00673*	**0** (0/55)	**100** (39/39)
*eogt^ex10^*	**16** (23/144)*	**99** (70/71)
eogt^ex10^	**30** (32/108)*	**95** (174/183)
*eogt* ^ex10^; *tub*-*EOGT*	**0** (0/104)	**0** (0/70)*
*eogt* ^ex10^; *tub-EOGT*	**0** (0/164)	**0** (0/180)*
*eogt* ^ex10^; *tub-Ago61*	**30** (11/118)^&^	**100** (82/82)
*dp^olvR^*	**58** (51/88)*	**100** (82/82)
*dp^olvR^*	**34** (38/113)*	**100** (157/157)
*dp^lv1^*	**22** (35/160)*	**99** (134/135)
*dp^v^; e(dp^v^)*	**0** (0/55)	**96** (108/112)
*pio^2R-1^*	**0** (0/45)	**100** (49/49)
*pio^MB03570^*	**0** (0/31)	**100** (34/34)
*pot^e04564^*	**0** (0/44)	**100** (31/31)
*mys^1^*	**0** (0/85)	**93** (86/92)
*crb^j1B5^*	**0** (0/113)	**85** (184/216)*
*wb^SF11^*	**12** (11/91)*	**100** (83/83)

*en>eogt^IR^* flies were crossed with indicated alleles. Three strains initially tested gave similar results so the data presented are from a single, triple recombinant *en>eogt^IR^* strain. The percentage of flies with wing blisters (n animals with blisters/total flies of appropriate genotype) is indicated. ^#^Baseline of two independent experiments; data for each allele were compared to appropriate control. *p<0.0001 or ^&^ *p*<0.02 by two-proportion Z-test in comparison to control.

Alleles of known *dp* interactors, as well as alleles encoding EGF repeat-containing proteins with blister phenotypes, were investigated for their ability to dominantly enhance or suppress the wing blister phenotype caused by *en*>*eogt^IR^*. *dp* mutants are classified according to three phenotypic classes: *dp^lethal^* (*dp^l^*) mutants carry null alleles and are homozygous lethal, *dp^oblique^* (*dp^o^*) alleles show an oblique wing, and *dp^vortex^* (*dp^v^*) alleles show vortices or commata of macrochaete on the thorax [26], [28]. As expected [11], we observed an enhancement with lethal *dp* alleles (*dp^olvR^* and *dp^lv^*) (Fig. 5E and Table 1). However *dp^v^*, a non-lethal vortex class allele of *dp,* in conjunction with the *dp^v^* enhancer *e^v^*, did not interact (Table 1). Wing blisters may arise due to separation of dorsal and ventral wing surfaces, and we therefore tested *mysopheroid* (*mys)*, a beta integrin linking the ventral and dorsal epithelia of the fly wing [29]. *mys^1^* did not interact with *eogt* in the wing (Table 1), and no integrin-like phenotypes were observed during embryogenesis or larval development of *eogt^ex10^* mutants. Nevertheless, removal of one copy of *wingblister* (*wb*), that encodes the ECM component laminin α chain [30], dominantly increased blister frequency (Table 1). Interestingly, laminin α has one EGF O-GlcNAc consensus site at T1799 and also contains a putative lectin domain, similar to the GlcNAc binding domain of the mammalian intermediate filaments desmin and vimentin [31]. Therefore, loss of O-GlcNAc due to *eogt* knock-down might lead to altered adhesive properties of laminins, and thus promote blistering.

Surprisingly, *pot* and *pio*, two well-documented *dp* genetic interactors implicated in the formation of the aECM [15], [16], gave no discernible interaction with *en*>*eogt^IR^* (Table 1). Neither misexpression of the EGF domains of the apical domain protein Crumbs (Crb) [32], which carries five O-GlcNAc consensus sites (EGF repeats 8, 10, 11, 13, and 26), nor absence of *crb* [33] are reported to result in a wing blister phenotype. Nevertheless, we detected weak but significant suppression of the *en*>*eogt^IR^* blister phenotype by loss of a *crb* allele (Table 1; see Discussion).

### Reduced Notch Signaling Suppresses Wing Blisters Due to *eogt* Knock-down

EGF repeats in N, Dl and Ser are substrates of EOGT (Figs. 1A, 1C and 1E). In addition, several independent *dp* alleles interact with the γ-secretase Presenilin (*psn*) [34], a crucial component of Notch pathway activation. However, embryos lacking both zygotic and maternal *eogt* do not show neurogenic phenotypes characteristic of *N* mutants ( [11]; and this work). In addition whereas overexpression of Ofut1, that transfers O-fucose to Notch EGF repeats, causes dramatic *N* phenotypes [35], *tubulin*-driven or restricted overexpression of *eogt* did not appear to affect Notch signaling in the wing or eye (not shown). However, effects of glycan removal on Notch signaling can be subtle, as observed in *rumi* mutants [36].

We therefore investigated interactions with Notch pathway mutants. Genetic interactions were detected with several mutant alleles of *N* including *Notch^Split^* (*N^Spl-1^*) with a point mutation in EGF14 [37], [38] that leads to an additional O-fucose site [39], *Notch^Abruptex^* alleles (*N^Ax16^, N^AxE2^, N^Ax9B2^*) [40] which harbor mutations in EGF24 or EGF29, and the loss-of-function *N^55E11^* allele. Each *N* mutant efficiently suppressed the blister phenotype (Table 2). The dominant L5 vein phenotype of *N^AxE2^* was not modified by knock-down of *eogt.* In addition, removal of one copy of several other Notch pathway members, including the ligands *Ser* and *Dl*, the transcriptional repressor *Su(H)*, and the transcriptional co-activator *mam*, also suppressed the *en>eogt^IR^-*induced wing blister phenotype by about 30% to 50%, indicating that Notch signaling promotes blistering due to loss of *eogt* (Table 2). Importantly, deficiencies uncovering these loci also suppressed wing blisters to corresponding degrees (Table 2).

**Table 2:** Notch Pathway Mutants Dominantly Suppress the Wing Blister Phenotype Induced by *en*>*eogt^IR^*.

Allele	% Flies with Blisters (22.5°C)	% Flies with Blisters (27°C)
*N^Ax16^*	**0** (0/155)	**52** (57/110)*
*N^AxE2^*	**1** (1/131)	**13** (26/197)*
*N^Ax9B2^*	**0** (0/132)	**35** (34/98)*
*N^Spl-1^*	**0** (0/88)	**40** (54/135)*
*Df(1)N-264-105*	**0** (0/78)	**41** (57/138)*
*N^55E11#^*	**0** (0/109)	**40** (54/135)*
*N^55E11#^*	**1** (1/93)	**37** (38/102)*
*Df(3R)ED6237* (*Dl*) *^#^*	**0** (0/80)	**41** (24/59)*
*Df(3R)ED6237* (*Dl*) *^#^*	**0** (0/71)	**48** (10/21)*
*Dl^RevF10^*	**0** (0/98)	**44**(45/103)*
*Df(3R)ED5942* (*Ser*)	**6** (7/124)	**70** (67/95)*
*Ser^RX106^*	**0** (0/92)	**68** (92/135)*
*Ser^RX106^*	**0** (0/120)	**76** (93/123)*
*Ser^RX82^& Dl^revF10^*	**0** (0/88)	**69** (81/118)*
*Df(3L)ri-79c* (*Psn*)	**0** (0/73)	**54** (72/134)*
*Psn^I2^*	**0** (0/165)	**40** (25/62)*
*Psn^227^*	**0** (0/105)	**52** (53/101)*
*Df(2L)TE35BC-4* (*SuH*)	**2** (2/113)	**80** (96/120)*
*Su(H)^2^*	**1** (1/148)	**73** (55/75)*
*Df(2R)BSC383* (*mam*)	**1** (1/130)	**43** (21/49)*
*mam^2^*	**0** (0/137)	**45** (52/116)*

*en>eogt^IR^* flies were crossed with indicated alleles. The percentage of flies with wing blisters (n animals with blisters/total flies of appropriate genotype) is indicated. ^#^Two independent experiments; data for each allele were compared to appropriate control. *p<0.0001 by two-proportion Z-test in comparison to control.

**Figure 6:**
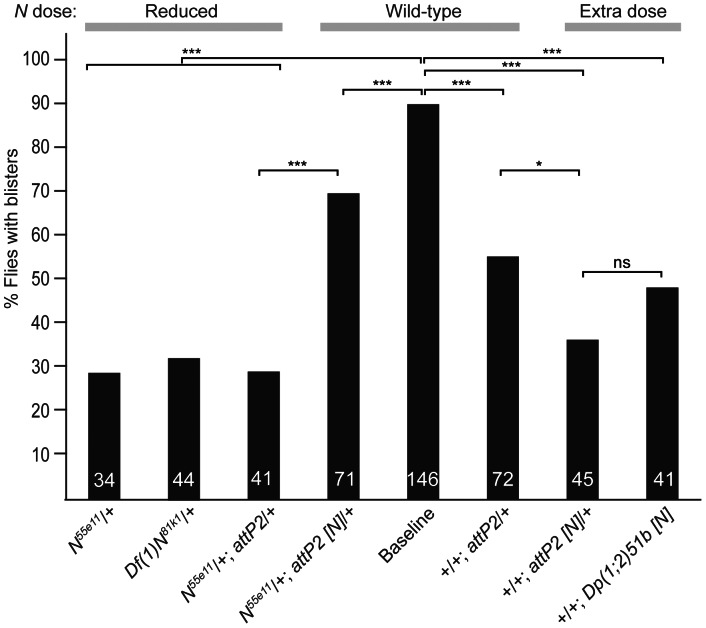
*N* gene dose influences wing blister frequency in *en>eogt^IR^* wings. The histogram depicts % animals with blisters of the genotype indicated below expressed in *en*>*eogt^IR^* (Baseline). *N* dose is indicated on top. Total number of animals of each genotype is shown at the base of each column. P-values were calculated using the Two-proportion Z-test. ***p<0.001; *p<0.05; ns, not significant.

**Figure 7:**
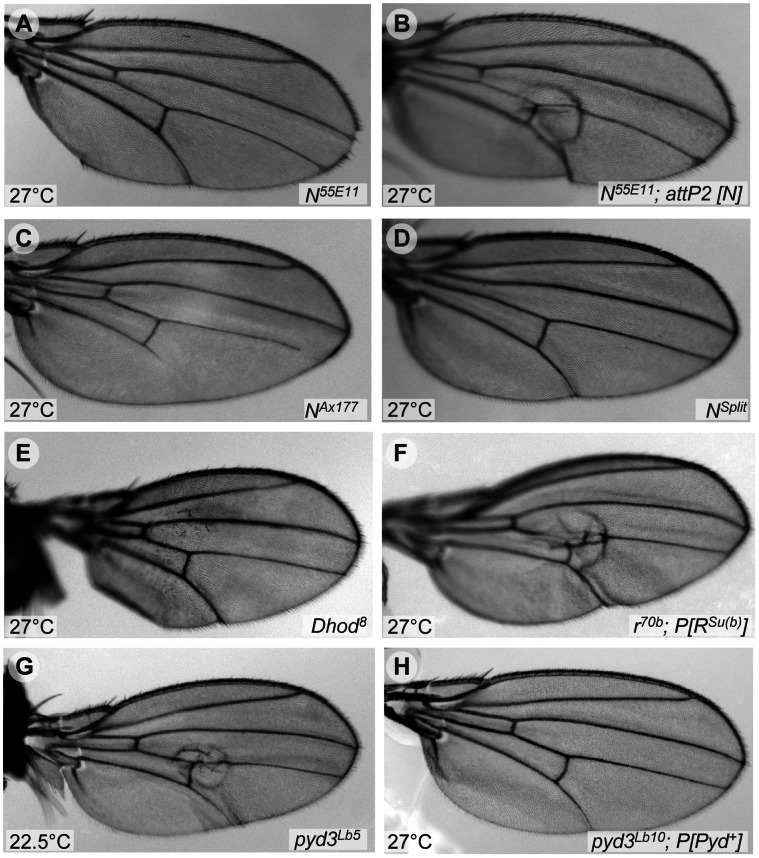
Wing blister phenotypes of *en*>*eogt^IR^* interactions with *N* and pyrimidine metabolism mutant alleles. Adult wings from flies with the *eogt^IR^* chromosome and the alleles shown developed at the indicated temperature. Several alleles of *N* suppressed the wing blister phenotype due to *eogt* knock-down (A, C, D). (B) *N^55E11^* suppression was reverted by a genomic *N* transgene. (E, H) Interactions of *en>eogt^IR^* with pyrimidine metabolism mutants. (E) *Dhod^8^* suppressed the wing blister phenotype of *en>eogt^IR^*. (F) The suppression of *en>eogt^IR^* by *r^70b^* was reverted by a transgene encoding constitutively active *R^Su(b)^.* (G) Example of a wing with blister from a fly with one null allele of *pyd3* (*pyd3^Lb5^*). (H) Example of a blistered wing of a fly overexpressing *pyd3* in an *en>eogt^IR^*; *pyd3^Lb10^*/+ background.

To verify that the suppression caused by *N^55E11^* was indeed due to a reduction of Notch activity, we attempted to revert suppression by adding back a dose of *N* from a genomic DNA construct integrated in the *attP2* site [41]. This construct in the *N^55E11^*/+; *en>eogt^IR^* background significantly reverted suppression of the blister phenotype caused by removal of one *N* allele (Figs. 6 and 7B). The reversion was not complete, however, presumably because the *attP2* control chromosome alone gave significant suppression (Fig. 6). Interestingly, an additional dose of *N* did not enhance the blister phenotype (Fig. 6), regardless of whether a Notch duplication *Dp(1;2)51b* or a *Notch* genomic transgene was used, but rather suppressed blister formation. This may be because flies with an additional *N* allele exhibit reduced Notch signaling in certain cell types (reviewed in [42]).

### Mutations in Pyrimidine Synthesis and Uracil Catabolism Modulate *eogt^IR^-*Induced Wing Blisters

**Figure 8:**
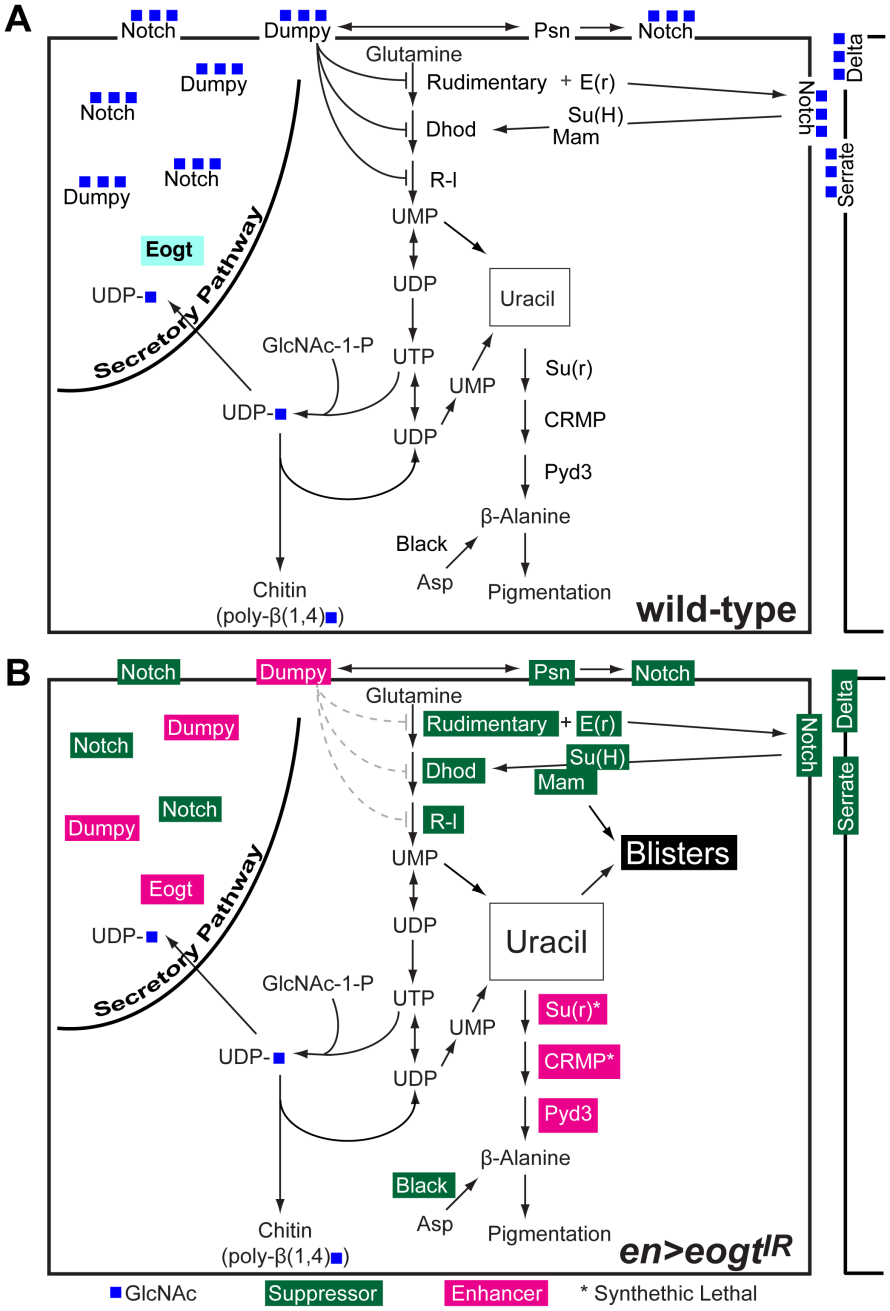
Interactions between Eogt, pyrimidine metabolism and Notch signaling in the posterior wing. (A) The diagram shows the Eogt-catalyzed addition of O-GlcNAc from UDP-GlcNAc (UDP-blue square) to EGF-containing proteins Dp and N, and key steps of the pyrimidine synthesis and catabolism pathways in a wild-type wing cell. Repression of pyrimidine neo-synthesis by Dp was shown biochemically in *dp* mutant larvae [17], [18]. Protein products of genes tested for interaction with *en*>*eogt^IR^* flies are indicated. (B) In *en*>*eogt^IR^* wings, reduced Eogt leads to loss of O-GlcNAc on Dp, N, Dl, Ser and other EGF-containing substrates. Genetic interactions with mutant alleles that resulted in suppression of wing blisters at 27°C are in green, while those that caused enhancement of wing blisters at 22.5°C are in magenta. Enhanced activity of initial enzymes in pyrimidine synthesis due to reduced Dp function is indicated by gray lines. The combined data suggest the unifying model that an increase in cytoplasmic uracil concentration is a likely cause of wing blisters when Eogt levels are reduced. The loss of O-GlcNAc from Dp and N may also contribute to the wing blister phenotype by reducing signals that influence pyrimidine biosynthesis.

Previous studies have shown that mutant *dp* alleles and the pyrimidine biosynthetic pathway functionally interact. Pyrimidine synthesis inhibitors fed to *dp^o^* mutant flies revert the oblique phenotype [43], and *dp* wing phenotypes are strongly enhanced in homozygous mutants in the pyrimidine catabolic enzyme *suppressor of rudimentary (su(r))* [19]. In addition several pyrimidine biosynthetic activities are increased in *dp* mutant larvae [17], [18]. We therefore tested mutations in enzymes of pyrimidine metabolism for modification of the *en>eogt^IR^* wing blister phenotype (Fig. 7 and Table 3). Pyrimidines are synthesized from glutamine (Gln) leading to the production of uridyl-derivatives including UTP, which is used in the synthesis of UDP-GlcNAc, the donor substrate of Eogt (Figs. 8A and S1). *De novo* biosynthesis is initiated by the multi-functional enzyme Rudimentary (R), followed by dihydroorotate dehydrogenase (Dhod), and a third enzyme Rudimentary-like (R-l), that encodes orotidine-5′-phosphate decarboxylase activity and generates UMP. Alleles of *r*, *Dhod* and *r-l* robustly suppressed the *en>eogt^IR^*-induced wing blister phenotype at 27°C (Fig. 7E for *Dhod^8^* and Table 3). When *en>eogt^IR^* flies were maintained at 31°C to further increase the expression of the RNAi transgene, *r* still suppressed the wing blister phenotype of *en>eogt^IR^* animals, albeit to a lesser extent, corroborating the dosage sensitivity of the interaction (Table 3). Importantly, we were able to partially revert the suppression of *en*>*eogt^IR^* by *r* in *In(1)r^70b^/+* animals (from 0% to 39% blisters) by rescuing *r* with a transgene encoding the UTP feedback-insensitive, thus hyperactive allele, *R^Su(b)^* [44], [45] (Table 3 and Fig. 7F). The wild-type *R* rescue construct was probably expressed at insufficient levels to revert the suppression (J. Rawls, personal communication). In addition, *enhancer of rudimentary* (*e*(*r*)), originally identified as a mutation that enhances the wing phenotype of *r* mutants [46], robustly suppressed the *en*>*eogt^IR^* phenotype (Table 3 *).* Therefore, mutations that cause a reduction in the synthesis of UMP suppressed the wing blister phenotype induced by knock-down of *eogt.*

**Table 3:** Mutants in Pyrimidine Metabolism Interact with *en>eogt^IR^*.

Mutant allele	% Flies with Blisters (22.5°C)	% Flies with Blisters (27°C)
*e(r)^G926^*	**0** (0/111)	**41** (31/76)*
*r^9^*	**0** (0/121)	**1** (1/114)*
*r^9^*		**54** (52/96)* $
*In(1)r^70b^; P{R}*	**0** (0/87)	**1** (1/99)*
*In(1)r^70b^; P{R^Su(b)^}*	**0** (0/124)	**39** (41/104)*
*Dhod^8^*	**0** (0/70)	**0** (0/81)*
*r-l^e01755^*	**0** (0/65)	**73** (84/115)*
*r-l^K2^*	**0** (0/84)	**2** (2/95)*
*su(r)^1^; b^1^*	synth. lethal	synth. lethal
*Df(3R)noi-B (CRMP)*	synth. lethal	synth. lethal
*b^1^; pyd3^Lb5^*	**3.5** (3/85)	**100** (98/98)#
*b^1^; P{PYD3^+^}; pyd3^Lb10^*	**0** (0/77)	**51** (67/132)*
*b^1^*	**0** (0/61)	**4** (3/85)*

*en>eogt^IR^* flies were crossed with indicated alleles. The percentage of flies with wing blisters (n animals with blisters/total flies of appropriate genotype) is indicated.

* *p*<0.0001 by two-proportion Z-test in comparison to control.

$ Cross was maintained at 31°C.

# 100% represents a significant increase (p<0.02) from the appropriate experimental series that had 95% baseline wing blisters (Table 1).

Since a reduction of pyrimidine neo-synthesis suppressed the *en*>*eogt^IR^* wing phenotype, we hypothesized that a reduction of pyrimidine degradation should enhance it. Pyrimidines are converted to uracil that is further metabolized to β-alanine (Fig. S1). We therefore tested loss-of-function alleles corresponding to pyrimidine catabolic enzymes as interactors. Dihydropyrimidine dehydrogenase, which metabolizes uracil to dihydrouracil and is encoded by *suppressor of rudimentary* (*su(r)*), has been shown to aggravate uracil toxicity in wild-type larvae and is highly 5-fluorouridine (5-FU) sensitive [47], [48]. Strikingly, and similar to *dp; su(r)* double mutants [19], removal of one gene dose of *su(r)* in the presence of *en*>*eogt^IR^* was lethal at 22.5°C, even though *su(r)* mutants are homozygous viable. Crosses of *su(r)* with control chromosomes lacking either the *eogt* dsRNA hairpin or the *en-Gal4* driver hatched, indicating that the observed synthetic lethality of *en>eogt^IR^* with *su(r)* was dependent on the expression of the *eogt* RNAi construct. We also examined the subsequent step, the conversion of dihydrouracil to 3-ureidopropionate by dihydropyrimidinase encoded by *Collapsin Response Mediator Protein* (*CRMP*; Fig. S1 *),* using the small deficiency *Df(3R)noi-B*, which deletes *CRMP* along with other genes [49], [50]. Again, this deficiency was synthetic lethal with *en>eogt^IR^.* Further downstream in the pathway of uracil catabolism, removal of one allele of *pyd3* led to a few flies with wing blisters at 22.5°C (Fig. 7G), as well as enhancement of the wing blister phenotype at 27°C (Table 3). Conversely, overexpression of wild-type *pyd3* from a transgene suppressed the wing blister phenotype of *en*>*eogt^IR^* from 100% to 51% at 27°C, even when one gene dosage of *pyd3* was removed in a *pyd3^Lb10^*/+ background (Fig. 7H), reflecting an increased metabolic flux towards β-alanine (Table 3). In *Drosophila*, β-alanine is also synthesized through decarboxylation of aspartate [51]. The *black^1^* (*b^1^*) mutation is a null allele for aspartate 1-decarboxylase (Fig. S1) [52], rendering pyrimdines the lone source of β-alanine in a *b^1^* background. We hypothesized that in the absence of *b*, more uracil would be metabolized to β-alanine, reducing uracil levels that might cause blisters. Consistent with this, the *b^1^* mutant suppressed the blister phenotype of *en>eogt^IR^* at 27°C (Table 3). The *b^1^* allele was also present in the *su(r)* and *pyd3* mutant stocks available, but it did not prevent the synthetic lethality of *su(r)* with *en>eogt^IR^,* consistent with the fact that mutants in pyrimidine catabolism are epistatic to the suppression of *b^1^* [48].

## Discussion

In this paper we identify a transcript of the human EGF-specific O-GlcNAc transferase EOGT that encodes O-GlcNAc transferase activity (Genbank KC347596.1). It is identical in sequence (527 aa) to the conceptual protein deduced from the proposed C3orf64 transcript b in AceView [53]. We show that transfection of this EOGT cDNA causes O-GlcNAcylation of *Drosphila* N, Dl and Ser EGF repeats; it also requires a conserved DXD motif for optimal activity, and it is primarily responsible for the transfer of O-GlcNAc to high molecular weight proteins, including Dp, in *Drosophila* larvae. Another cDNA in Genbank (NM_173654.1; [21]) lacks an internal segment that contains the DYD motif, and is therefore predicted to have low or no activity. *In vivo,* the human *EOGT* cDNA fully rescued homozygotes of a new P-element excision mutation of *eogt* (*eogt^ex10^*). Both *eogt* RNAi knock-down and mutant clones exhibited blistered wing and vortex phenotypes, similar to the vortex class of *dp* mutants (*dp^v^*). The hallmark of the removal of *eogt* in the posterior wing in *en>eogt^IR^* flies was the temperature-dependent development of blisters, with essentially 100% frequency at 27°C (Table 1 and Fig. 5; [11]). Importantly, wing blister formation was enhanced at 22.5°C by the *eogt^ex10^* mutation, and fully rescued at 27°C by the human *EOGT* transgene, but not a transgene encoding the related mouse gene *Ago61* (Table 1).

To investigate the potential origin of *en>eogt^IR^*-induced wing blisters, we used a candidate genetic interaction strategy. The reduction in O-GlcNAc transfer caused by loss of *eogt* physically affects EGF-repeat containing proteins of the secretory pathway. Thus, we examined genetic interactions with mutants of *Notch*, *crumbs*, *dumpy* and *wingblister* (Laminin α), which all contain EGF repeats with the consensus site for recognition by Eogt. We also investigated integrins known to cause wing blisters. If a reduction in O-GlcNAc caused by *en>eogt^IR^* affects function due to loss of activity, stability, or altered localization of a membrane glycoprotein, the development of a wing blister may be a direct consequence. In that case, the additional loss of one dose of that glycoprotein should further reduce activity, and thus enhance blister formation due to *en>eogt^IR^* knockdown. If, on the other hand, activity is not altered by the loss of O-GlcNAc, the consequences of removal of one allele of a substrate would be independent of O-GlcNAc status. Alternatively, the lower amount of protein might not be limiting, and thus no dosage sensitivity would be observed. If protein function was enhanced by the loss of O-GlcNAc, leading to promotion of blister formation, removal of one allele should suppress blister formation.

In the case of Dumpy, a very large protein of the aECM [13], reduced O-GlcNAc on Dp EGF repeats might cause a failure of wing integrity due to loss of O-GlcNAc-mediated cell adhesion, perhaps via a partner lectin like the laminin Wingblister, thereby disrupting cell-matrix-chitin interactions. This would be consistent with enhancement of the *en>eogt^IR^* wing blister phenotype by lethal alleles of *dp* (Table 1 and Fig. 8B; [11]). Unxepectedly, mutant alleles of the confirmed *dp* interactors *pio* and *pot* [15], [16] did not interact in our assay (Table 1), even though wing clones of *pio* and *pot* give rise to blisters [16]. The integrin *mys^1^* also did not enhance the phenotype in the posterior wing (Table 1; [11]). This might indicate that O-GlcNAc on Dp is not required to mediate interactions between *dp* and those genes, or that loss of one allele of those genes is not sufficient to expose a genetic interaction. By contrast, loss of one allele of the Laminin α chain *wb* enhanced the *en>eogt^IR^* wing blister phenotype, potentially because the loss of O-GlcNAc from the Wingblister EGF repeat [30] reduces its activity. Thus, the wing blisters formed in *en>eogt^IR^* wings may arise in part from the loss of O-GlcNAc on Dp and Wb. However, the origin of the wing blisters must be more complex because of genetic interactions detected with mutants in the Notch signaling pathway, and with mutants in pyrimidine biosynthesis and catabolism that occur in the cytoplasm and mitochondria.

In the case of Notch, suppression of the *en>eogt^IR^* wing blister phenotype was observed in a *N^55E11^*/+, *N^Ax^*/+ or *N^Spl^*/+ background (Table 2). Importantly, the suppression observed in *N^55E11^*/+ heterozygotes was largely reverted by the addition of a genomic copy of *N,* the product of which would carry little if any O-GlcNAc in an *en>eogt^IR^* wing. Thus, Notch signaling may promote blister formation whether or not it carries O-GlcNAc, so that loss of Notch signaling would suppress blister formation. This conclusion is consistent with the observations that mutations in *Ser*, *Su(H)*, *Dl* and *mam,* as well as deficiencies of these alleles, suppressed *en>eogt^IR^*-induced blister formation (Table 2). Unfortunately, expression of *N^ICD^* or *N^ΔECD^* in the *en>eogt^IR^* background were both lethal, and it was thus not possible to assess the effect of constitutively active N on *en>eogt^IR^* induced blister formation. Similarly, balanced compound heterozygotes of *N* and *dp^l^* alleles did not produce offspring, preventing us from assessing if removal of one *N* allele suppresses blisters of *dp^l^* clones. Although only one mutant allele of *crb* was investigated, the slight suppression in wing blister formation obtained upon removal of one *crb* allele may be due to relief of Crb inhibition of Presenilin-induced Notch activation [33], rather than to loss of Crb regulation of epithelial apical–basal polarity [54].

A potential unifying hypothesis that may tie defective Dp function and Notch signaling to opposite effects on the development of wing blisters in *en>eogt^IR^* flies, is that both pathways interact with the pyrimidine synthesis pathway. Biochemical data show that at 72 hr several *dp* mutants have increased aspartate transcarbamylase (ATC; one of the activities encoded by *r*), orotate phosphoribosyltransferase (OPRT) and orotidine-5′-phosphate decarboxylase (ODC) activities, both encoded by *r-l* [17], [18] (Fig. S1). In addition, mutations in *r* that decrease ATC activity, suppress the development of *dp* mutant wing phenotypes, i. e. they normalize truncated oblique (*dp^o^*) mutant wings [17]. Administration of the ATC inhibitors 6-azauracil and 6-azauridine to inhibit pyrimidine synthesis, causes phenotypes that mimic *r* but normalize *dp* phenotypes [43], [55]. Consistent with the fact that loss of one allele of *dp* enhances *en>eogt^IR^* blisters, loss of one allele of *r* suppresses the formation of these blisters (Table 3; Fig. 7). In fact, complete and revertable suppression of the *en>eogt^IR^* wing blister phenotype was obtained with several mutants in pyrimidine neo-synthesis, including *r, r-l* and *Dhod.* The suppression of wing blister formation by reduced pyrimidine biosynthesis, along with the synthetic lethality (that we interpret as enhancement) observed when uracil is not removed by catabolism in *en>eogt^IR^* flies, suggest that increased levels of uracil promote or cause blister formation. Thus, the interaction of *en>eogt^IR^* with *su(r)* recapitulates a strong enhancement of *dp* wing phenotypes by *su(r)* that leads to blistered wings [19]. Also consistent is that the pyrimidine catabolic pathway activator *black* suppresses the *en>eogt^IR^* blister phenotype (Table 3; Fig. 8). On the other hand, flies homozygous for certain alleles of *r* can develop wing blisters as part of a vastly smaller wing, in spite of reduced pyrimidine synthesis [48]. The multiple mechanisms of wing blister formation are clearly varied and complex. In this context, it is worth mentioning that the reduction in wing size of *dp* and some *r* mutants have different origins, with the first being due mainly to changes in cell size [56], and the latter predominantly to a reduction in cell number [57].

Taken together, the facts that Dp is a substrate of Eogt, that *dp* and *eogt* mutants phenocopy each other, and that both exhibit genetic interactions with pyrimidine biosynthesis mutants, suggest a model in which Dp-O-GlcNAc slows the production of pyrimidines, while reduction of Dp or Dp that lacks O-GlcNAc enhances *de novo* pyrimidine synthesis. Consequently, overproduction of a toxic UMP metabolite such as uracil leads to the *eogt* RNAi blister phenotype (Fig. 8). This uracil toxicity model does not discount Dp-mediated, adhesion-dependent mechanisms of action during wing development. Due to chitin synthesis, UDP-sugar concentrations in the hemolymph of insect wing discs are enormous [58], and a corresponding amount of UDP liberated by chitin synthases in a short time might well upset baseline pyrimidine synthesis regulation, and require a temporally active, tissue specific regulatory mechanism.

The involvement of the EGF-O-GlcNAc modification in pyrimidine synthesis is consistent with published data on *dp* mutants. In contrast, the fact that decreased *Notch* pathway activity suppressed the *en*>*eogt^IR^*-induced blister phenotype (Table 2; Fig. 5) was unexpected and counterintuitive, given that *psn* and *dp* loss-of-function alleles collaborate in the formation of a *dp* vortex phenotype on the thorax [34], and that *Dl* and *mam* alleles can present wing blisters [26]. The strong suppression observed with *N* alleles affected only in EGF repeats (*N^Ax^* and *N^Spl^*) might be consistent with a decrease in a putative O-GlcNAc-dependent, Notch ligand-induced signal important for the generation of blisters (Fig. 8B). The *N^Ax9B2^* mutant has a mutation in EGF24, next to EGF25 that has an Eogt consensus site, and also suppressed the blister phenotype. Interestingly, two different *Ax* point mutations in EGF29 of Notch (SHVC_4_YC_5_SQGYAGSYC_6_Q to SHVC_4_YC_5_SQAYAGSYC_6_Q in *Ax^16^* [38], or to SIVC_4_YC_5_SQGYAGSYC_6_Q in Ax^E2^ [37], [38]), suppressed the RNAi phenotype somewhat dissimilarly. The *Ax^E2^* allele was the best suppressor of the *en*>*eogt^IR^* blister phenotype in the Notch pathway series of interactors, suppressing blister frequency by 87%. It is surprising that this *Ax* allele gave stronger suppression than a *N* deficiency. Suppression through either loss- or gain-of-function *N* alleles, may be directly due to diminished Notch signaling. Consistent with the first possibility, humans with homozygous mutations in *EOGT* [59], or autosomal dominant mutations in RBPJ (CSL homologue of *Su(H)*) [60]), a downstream effector of Notch signaling, have been shown to cause Adams-Oliver Syndrome, a developmental disease with limb abnormalities and skin defects. However, it is also possible that suppression is indirect and due to attenuation of *de novo* pyrimidine synthesis (Fig. 8). Notch signaling and pyrimidine metabolism have recently been shown to converge at the *e(r)* locus. Mutations of *e(r)* are homozygous synthetic lethal with otherwise viable and mild *N* mutations that also interact with *Dl* but not *Ser* [61]. *e*(r) is a nuclear protein [62] that promotes pyrimidine production [46], and an *e(r)* mutant suppressed the wing blister phenotype to the same degree as *N* or *Dl* null mutants (Table 3). Earlier work [63] also indicates an ∼50% decrease of Dhod enzyme activity in larvae heterozygous for a *N* loss-of-function mutation, indicating further involvement of *N* in pyrimidine synthesis regulation (Fig. 8).

In summary, our combined data suggest that an increase in cytoplasmic uracil concentration is a likely cause of wing blisters when Eogt levels are reduced (Fig. 8). In addition, the loss of O-GlcNAc from Dp and N may also contribute to the wing blister phenotype, for example by reducing signals that affect pyrimidine metabolism and increase uracil levels. A genome wide screen for *eogt* interactors could reveal lectin(s) or ligands that interpret the O-GlcNAc signal on EGF repeats, and the signals to mitochondria (*Dhod*), the cytosol (*r, r-l*) and possibly the nucleus (*e(r)*) that regulate pyrimidine synthesis.

## Materials and Methods

### Antibodies

Polyclonal mouse anti-human EOGT (AER61) was from ABCAM (ab69389). Mouse monoclonal anti-O-GlcNAc IgM (CTD110.6; O7764), rabbit anti-mouse Ago61 (AV48972), and anti-α-tubulin (T5168) were from Sigma. Rabbit anti-GFP was from Invitrogen (A11122). Goat anti-human PLAP was from Santa Cruz (L-19, sc-15065) and mouse anti-His was from Roche (#11922416001). All antibodies were diluted in 3% bovine serum albumin (BSA) in Tris buffered saline pH 7.4 (TBS), and 0.1% Tween 20.

### Plasmids

pMT-Notch(EGF1-20-AP), pMT-WB-Delta-AP-6His, pMT-WB-Serrate-AP-6His were a kind gift from Ken Irvine (HHMI and Waksman Institute, Piscataway, NJ). pCaspTubPA was a kind gift of Stephen Cohen (Institute of Molecular and Cell Biology, Singapore). pCMV-SPORT6 mouse Ago61 (MMM1013-7512204) was purchased from Open Biosystems (Thermo Scientific). *Drosophila eogt* cDNA (GH04522) in pOT2 was obtained from DGRC (Indiana University, In). Human *EOGT* cDNA was cloned from HEK 293T cells that were extracted with Trizol (Invitrogen) to obtain total RNA from which polyA^+^ RNA was purified with the Genelute mRNA Miniprep Kit (Sigma) according to the manufacturer’s recommendations. Reverse transcription was performed using Superscript III reverse transcriptase (Invitrogen). *EOGT* was amplified using primers PS1427 and PS1428 (Table 4) and cloned into pCR2.1TOPO (Invitrogen). For this study, we used an isoform that was active in GlcNAc transfer and had an amino acid sequence identical to chimp *Eogt* (NP_001009171). The Genbank accession number is KC347596.1. Chinese hamster *Eogt* cDNA was amplified by RT-PCR of total RNA from Chinese hamster ovary cells (CHO; clone Pro^-^5 and two independent clones) using primers PS1271 and PS1166r (Table 4). The Genbank accession number is KC347595.1.

**Table 4:** Oligonucleotide Primers.

PS1427	h*EOGT*	GAGGTTTGCAGGTTGCATGT
PS1428	h*EOGT*	GTCTGGGTGTTGGAGTGTTT
PS1271	CHO *Eogt*	ACTWARAGGGTCTGCAGGTTGCT
PS1166r	CHO *Eogt*	TCTGCAGCCTGMAGGACAAG
PS1444	m*Ago61*	ATAAGAATGCGGCCGCAAAAATGCACCTCTCTGCCGTATTC
PS1448	m*Ago61*	CCGCTCGAGCTACGTGCTGCACACCAGCACAT
PS1450	CG9867	ATAAGAATGCGGCCGCCAAAATGCCAATCCTGCCAATACTC
PS1452	CG9867	CCGCTCGAGCTACAGCTCGTTGCGCTGCGTTTTGG
PS1446	h*EOGT*	ATAAGAATGCGGCCGCCAAAATGTTAATGTTGTTTGTCTTTG
PS1449	h*EOGT*	CCGCTCGAGTTATAGCTCATCATGTTTCTTC
PS1550	AYA N-term	GGAGATCTGGTCAGAATGAAGCTCCTCCTAATACTCACAGCA
PS1454	AYA N-term	CAAATGTATAAGGCATAAGCAGTAAATGC
PS1453	AYA C-term	GCATTTACTGCTTATGCCGTTATACATTTG
PS1551	AYA C-term	GGTCTAGATTTATAGCTCATCATGTTTCTTCTTAAATG
PST71429	*eogt* dsRNA	*TAATACGACTCACTATAGGG* AGACCACGGGAACATACCGGCTGGGCC
PST71430	*eogt* dsRNA	*TAATACGACTCACTATAGGG* AGACCACCGATTTTCATGATGAAAGTGGG
PS1378	*eogt* excision site	GCATGCCGATGAGTATG
PS1380	*eogt* excision site	CAATAACATTATCCCGCTCAT
345for	*sxc*6 point mut.	GCTGAATGCACCGACCAACGCTGTGGACAC
345rev	*sxc*6 point mut.	GAACGATAAAGTCAAGATGTAGAGCGAC

Upper and lower sequences are forward and reverse primers, respectively. Italicized bases are T7 extensions.

PCR products of full-length mouse *Ago61* obtained with PS1444 and PS1448, human *EOGT* (PS1446 and PS1449) and *Drosophila eogt* (PS1450 and PS1452) coding sequences were cloned into pSC-A vectors (Agilent) introducing a 5′ *Not*I site and a ‘CCACC’ Kozak sequence [64] and a 3′ *Xho*I site (Table 4). They were further subcloned into pMT-V5/His-A (Invitrogen) and pCaspTubPA. pUAST*eogt* was made by inserting *eogt* as a *Bgl*II and *Xho*I fragment from pOT2 GH04522 into pUAST [65]. Genetic Services, Inc. (Cambridge, MA) injected DNA transgenes.

To identify a DXD motif conserved across species, Eogt sequences were compared using CLUSTAL W [66]. Accession numbers for Eogt from the respective species were: Chinese hamster ovary cells (CHO Pro^-^5): KC347595.1; *Trichoplax adhaerens:* XP_002117650.1; *Drosophila melanogaster:* NP_608678.1; *Ciona intestinalis*: NP_001027841.1; *Caenorhabditis elegans*: NP_506677.3; Family 61 protein from *Arabidopsis thaliana*: NP_565952.1; DUF563 protein from *Cyanothece sp*. PCC 7425: YP_002485842.1.

Site-directed mutagenesis of human *EOGT* to change DYD to AYA was performed by overlap extension PCR [67] using primers PS1550 and PS1454, for the N-terminus and primers PS1453 and PS1551 for the C-terminus (Table 4). Wild-type *EOGT* was amplified using PS1550 and PS1551. PCR products were digested with *Bgl*II and *Xba*I and cloned into the *Bgl*II/*Spe*I sites of pMT-Bip-V5/HisA (Invitrogen) in frame with the Bip signal sequence. Mutations were confirmed by DNA sequencing.

### Cell Culture

S2 cells were cultured in *Schneider’s Drosophila Medium* (Invitrogen, CA) supplemented with 10% heat inactivated fetal calf serum (Gemini Bio Products, CA) at 25°C. Transient transfection with Ca_2_PO_4_ was carried out following the Invitrogen *Drosophila* Expression System manual protocol for transient expression using 20 µg plasmid DNA per 35 mm dish. Where appropriate, protein expression was induced with 1 mM Cu_2_SO_4_ and cells and medium harvested after 24 h or 48 h at 25°C.

To generate RNAi constructs that targeted *eogt,* DNA template corresponding to VDRC/44572 (Fig. 2A) was generated from *w^1118^* genomic DNA by PCR with primers PST71429 and PST71430 (Table 4), containing a T7 promoter at each end. Products were cloned into pSC-A (Agilent) to create pSC-A-T7-44572-T7. A gel-purified *EcoR*I fragment from the plasmid pSC-T7-44572-T7 was used as template for PCR with the same primers, products were gel-purified using the QIAquick Gel extraction kit (Qiagen), and used as templates for *in vitro* transcription reactions using a Megascript RNAi kit (Ambion) following the manufacturer’s instructions. The dsRNA was purified using Qiagen’s RNeasy kit following the manufacturer’s protocol except that β-mercaptoethanol was omitted. S2 cells (2.2×10^7^) were seeded onto a 10 cm dish in S2 medium. After cells adhered, the medium was replaced with serum-free medium, 200 µg of dsRNA or water was added, and the dish shaken every 20 min. After 1 h, heat-inactivated serum was added to 10% final concentration. On day 5 after treatment, the cells were split 1∶4 into new 10 cm plates, and the dsRNA treatment was repeated. On day 9 of dsRNA treatment, the cells were transfected with 10 µg appropriate plasmid DNAs using Ca_2_PO_4_. Post nuclear supernatant was obtained after lysis in TBS, 1% TX-100 and a 5 min centrifugation step at 1000 rpm at 4°C. Protein lysate (50 ug) was separated on a 10% SDS-PAGE and 6.5% SDS-PAGE (to resolve the anti-Ago61 signal) with a 4% stacking gel. Immunoprecipitates to analyze secreted proteins were obtained by incubating 4 ml S2 conditioned medium with 280 µl of 10× *Complete* Proteinase Inhibitor (Roche), 400 µl of 1.5 M NaCl, 80 µl of 1M Tris-HCl (pH 7.4), 20 µl anti-PLAP beads from Sigma (A2080). Beads were washed 3 times using TBS with 1% TX-100 and 50% of the immunoprecipitated sample was loaded onto the SDS-PAGE.

### Protein Extraction and Gel Electrophoresis

For Western analysis, *sxc^6^* and *eogt^ex10^* mutants were balanced over CyO-*twi-Gal4>UAS-GFP* [68]. Embryos were collected for 2 hours on apple plates. At 60–62 h AEL, GFP-negative larvae were selected, flash frozen and stored at −80°C. Larvae (100 per genotype) were homogenized for 50 sec using a tissue homogenizer (Kontes) in PBS lacking Ca^2+^ and Mg^2+^ and containing 100 µM of the Ogt inhibitor PUGNAc (Sigma) and 1× *Complete* Proteinase Inhibitor (Roche). Samples were centrifuged twice at 3000 g for 5 min and the supernatant transferred to a new tube.

*tub-Gal4/*TM3,*Sb,act-5C-GFP* flies were crossed to homozygous *UAS-dp^VDRC44029^* flies and the embryos were aged on apple plates at 31°C to ensure efficient knock-down. After 5 days, 7 GFP-positive (controls) and 7 GFP-negative (*dp* knock-down) pupae were lysed and processed as above, except that lysates were centrifuged at 1000 g for 20 min at 4°C. Pupae extract (∼100 µg protein) was separated on a 7.5% SDS-PAGE with a 3.5% stacking gel and processed for Western analysis.

### Fly Stocks and Transgenic Lines

*Drosophila* RNAi lines *UAS-eogt^VDRC44572^* and *UAS-dp^VDRC44029^* were obtained from the VDRC (Vienna, Austria) and *UAS-eogt^R3^* (9867R-3) was obtained from the National Institute of Genetics (Mishima, Japan). Other lines were obtained from the Bloomington Stock Center. *en-Gal4*, *heat-shock-Gal4*, *ap-Gal4*, *act-Gal4*, and *tub-Gal4* are described in Flybase (http://flybase.org/). *eogt* mutants were generated by imprecise P-element excision of isogenized *BG00673* (Bloomington Stock Center). Excision mutant *eogt^ex10^* is homozygous lethal and lethal over *Df(2L)Exel7011*, a deficiency uncovering *eogt*. The region around the P-element excision site was PCR amplified with primers PS1378 and PS1380 (Table 4), and sequenced using DNA from viable *eogt^ex10^*/*BG00673* adults. The excision induced a deletion of 171 bp upstream of the ATG to 673 bp downstream of the ATG (conceptually deleting 224 aa, including the start codon; Fig. 2A).

Mutant wing and thorax clones were generated after recombination of *dp^lv1^* and *eogt^ex10^* onto Frt40A chromosomes using standard procedures. Recombinants were screened for the presence of the alleles by backcrossing to *dp* ^olvR^ and *Df(2L)Exel7011*, respectively. In the case of *eogt*, recombinants were confirmed by PCR. Unmarked clones were generated using *y w Ubx-flp*. *sxc* ^6^, *eogt* ^ex10^ recombinants were tested by backcrossing separately to *sxc* ^6^ and *eogt* ^ex10^ and confirmed by PCR with primers PS1378 and PS1380 (Table 4). The presence of the point mutation in the *sxc* ^6^ allele [9] was confirmed by sequencing a PCR product obtained with primers 345for and 345rev (Table 4).

To test for genetic interactions, an *en-Gal4*, *UAS-VDRC^44572^*, *UAS*-*dcr2*/CyO triple recombinant strain (here called *en>eogt^IR^*) was generated, by first recombining *UAS*-*dcr2* with *UAS*-*VDRC^44572^*. The *en-Gal4* driver was then recombined onto the same chromosome and recombinants were identified by their ability to induce wing blisters over a CyO chromosome that contained a *dp* ^l^ mutation at 25°C. Initially, three independent recombinants were compared and showed similar results. Data presented here are derived from one of these recombinants. The following stocks were used for genetic interaction tests (Numbers in brackets correspond to Bloomington stock numbers): *dp* ^olvR^/*SM5* (280), *dp^lv^, b^1^*/*SM5* (278), *dp^v^; e(dp^v^)^1^* (690), *N^co^ FRT18/*FM6, *yw*; *crbj^1BS^*/TM3 (10331), *w^1118^, PBac* [*55*] *fw^e04564^*/*FM7c* (18273); *y^1^,w^1^;FRT4*2D *pio^2R-16^ P{white-un1}47A*/CyO, *P{w^+mC = act-lacZ.B^}CB1* (2278), *w^1118^; Mi{ET1}CG10513^MB07190^* (25543), *yw*; *FRT82B Dl^rev10^*/TM6B, *w*; *FRT82B Ser^RX106^*/TM6B, *N^55e11^ rb* ^1^/*C(1)DX*, *y* ^1^ *w* ^1^ *f^1^*; *Dp(1;2)51b*/+ (3015), *Df(1)N-264-105/FM1*, *lz^+^* (a deficiency uncovering *Notch*, 731), *mys^1^*/FM4 (59), *wb^SF11^Adh^UF^ cn* ^1^/CyO (3409), *Dl^RevF10^*, *Ser^RX82^ FRT*82B/TM6B, *Tb* ^1^ (6300), *N^spl-1^* (118), *w^1118^*; *Df(3R)ED5942/*TM6C, *cu^1^ Sb^1^* (uncovering *Ser*, 8922), *w^1118^*; *Df(3R)ED6237/*TM3, *Ser^1^* (uncovering *Dl*, 9280), *w* ^a^, *N^55e11^*/*FM7c, yw, Ax^16^/yw, Ax^16^, y, Ax^E2^/y, Ax^E2^, Ax^9B2^, sn^3^/Ax^9B2^, sn^3^*, *Df(3L)ri-79c/*TM3, *Sb^1^* (a deficiency uncovering *presenilin*, 3127), *Psn^I2^*/TM6C, *Sb^1^* (5463), *Psn^227^*/TM6B, *Tb^1^* (8300), *pr^1^ cn^1^ Su(H)^2^*/CyO (30477), *Df(2L)TE35BC-4*, *b^1^ pr^1^ pk^1^ cn^1^*/CyO (uncovering *Su(H)*, 6088), *mam^8^*/CyO (1596), *cn^1^ mam^2^ bw^1^ sp^1^*/CyO (3983), *w^1118^*; *Df(2R)BSC383/*CyO (uncovering *mastermind*, 24407), *r^9^*/C(1)DX, *y^1^ f^1^* (83), In(1)*r^70b^*, w^*^ r^70b^; *P{w^+mC = rcSa^}9*/+ (24177), In(1)*r^70b^*, w^*^ r^70b^; P*{w^+mC^ = r^Su(b).cSa^}*6/+ (24178), *su(r)^1^ rC*; *b* ^1^ (5822), w^*^ *P{^w+mC = EP^} e(r)^G926^* (33462), *Dhod^8^*/TM3, *Sb^1^* (2571), w^*^; b^*^; CRMP^supI2^/TM3, *Sb^1^ Ser^1^* (24173), *Df(3R)noi-B*: derivative of *p[W+]Pyd2* (*CRMP*); *b^1^*; *Df(3R)noi-B*, e1 (24479) after removal of the *p[W+]Pyd2* (*CRMP*) transgene, w^*^; *b^1^*; *pyd3* ^Lb5^/TM3, *Sb^1^ Ser^1^* (24175), w*; P*{w^+mC = PYD3+^}3b b^1^*; *pyd3^Lb10^*/TM3, *Sb* [*1*] *Ser* [*1*] (24176).

To vary the dosage of *N*, we used a genomic transgene of *N* integrated in *attP2* (*y w*; *Notch^gt-wt^*::*attP2* [69]) and an empty *y^1^ w^67c23^*; *PattP2* [41] chromosome as control. Males of *Df(1)N-81k1*, *dnc^81k1^*/Y; SM1, *Dp(1;2)51b*/+ (3006) were crossed to *en>eogt^IR^* virgins to obtain females with one copy of functional *N* and a *Notch* specific deficiency. A two proportion Z-test online http://in-silico.net/tools/statistics/ztest/two-proportion was used to assess the statistical significance of an interaction.

## Supporting Information

Figure S1 **Pyrimidine anabolic and catabolic pathways.** The IUBMB names of pathway enzymes in pyrimidine biosynthesis and catabolism are shown with the product generated by each reaction. Green bars signify steps for which reduced enzyme activity caused suppression of wing blisters in *eogt^IR^* wings; magenta bars signify steps for which reduced enzyme activity caused enhancement of wing blisters in *eogt^IR^* wings.
(TIF)
