# Supplementary figures and images for: The EGF Repeat-Specific O-GlcNAc-Transferase Eogt Interacts with Notch Signaling and Pyrimidine Metabolism Pathways in Drosophila
Source: PLoS One. 2013 May 9;8(5):e62835. doi: 10.1371/journal.pone.0062835 (PMC3650022; doi:10.1371/journal.pone.0062835)

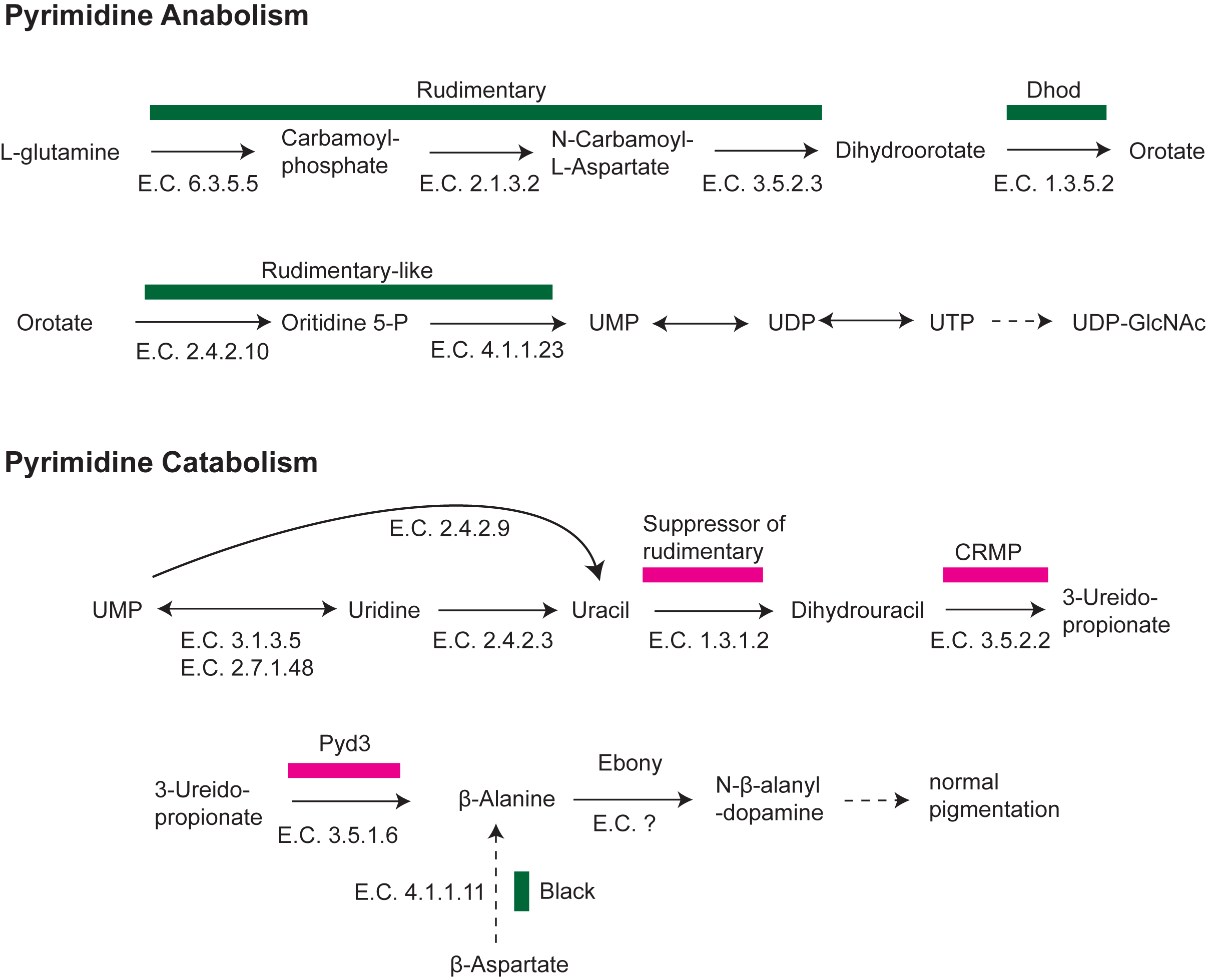

Supplement: Figure S1 — Pyrimidine anabolic and catabolic pathways. The IUBMB names of pathway enzymes in pyrimidine biosynthesis and catabolism are shown with the product generated by each reaction. Green bars signify steps for which reduced enzyme activity caused suppression of wing blisters in eogtIR wings; magenta bars signify steps for which reduced enzyme activity caused enhancement of wing blisters in eogtIR wings. (TIF) [file pone.0062835.s001.tif]
